# Supplementary material for: Unlocking osmotic energy harvesting potential in challenging real-world hypersaline environments through vermiculite-based hetero-nanochannels
Source: Nat Commun. 2024 Jan 19;15:608. doi: 10.1038/s41467-023-44434-1 (PMC10799064; doi:10.1038/s41467-023-44434-1)
Supplement: Supplementary file 1 — Supplementary Information [file 41467_2023_44434_MOESM1_ESM.pdf]

Supplementary Information for

Unlocking Osmotic Energy Harvesting Potential in Challenging Real-World Hypersaline Environments through Vermiculite-Based Hetero-Nanochannels

Jin Wang[1, *], Zheng Cui[1], Shangzhen Li[1], Zeyuan Song[1], Miaolu He[1], Danxi Huang[1], Yuan Feng[1], Yanzheng Liu[1], Ke Zhou[2, *], Xudong Wang[1], Lei Wang[1, *]

Correspondence to: wangjin@xauat.edu.cn; zhouke@suda.edu.cn; wl0178@126.com

[1]Research Institute of Membrane Separation Technology of Shaanxi Province, Key Laboratory of Membrane Separation of Shaanxi Province, School of Environmental & Municipal Engineering, Xi'an University of Architecture and Technology, No. 13 Yan Ta Road, 710000 Xi'an (China).

[2] College of Energy, Soochow Institute for Energy and Materials InnovationS (SIEMIS), Jiangsu Provincial Key Laboratory for Advanced Carbon Materials and Wearable Energy Technologies, Soochow University, 215006 Suzhou (China).

*Correspondence to: wangjin@xauat.edu.cn; zhouke@suda.edu.cn; wl0178@126.com.

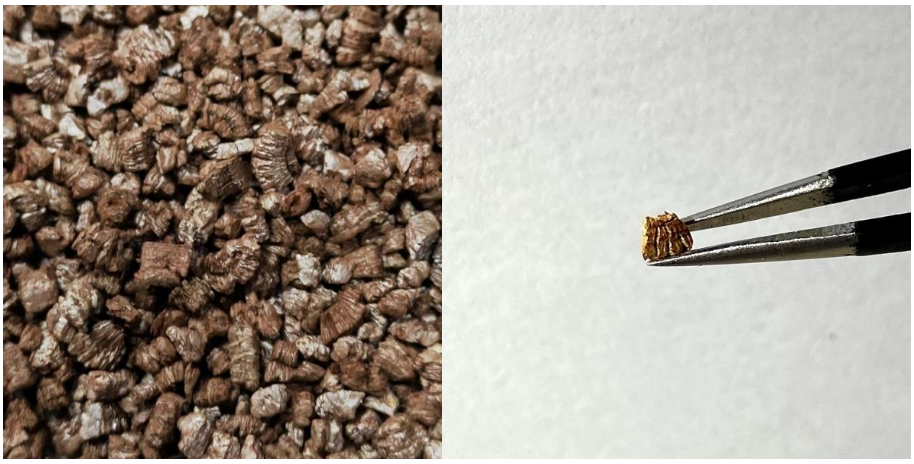

Supplementary Figure 1. Optical images of the raw thermally expanded vermiculite (VMT).

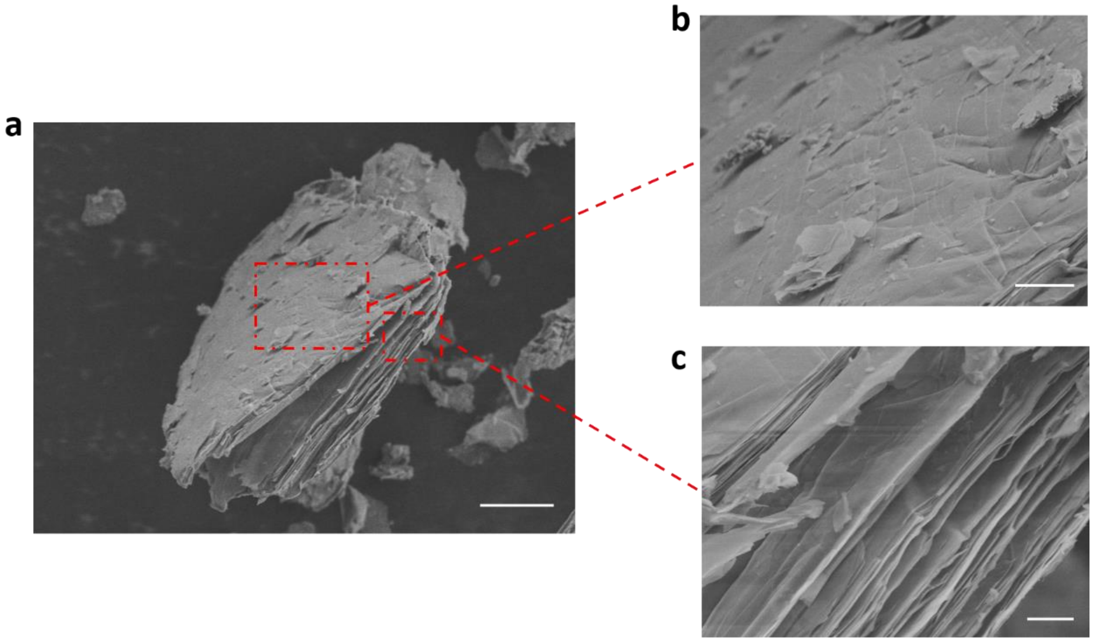

Supplementary Figure 2. SEM images of the raw thermally expanded VMT particle. The raw VMT particle showed aligned and multilayered structural characteristics. Scale bar, **a**, 20 μm, **b**, 5 μm, **c**, 2 μm.

Day 0 Day 14 Day 30

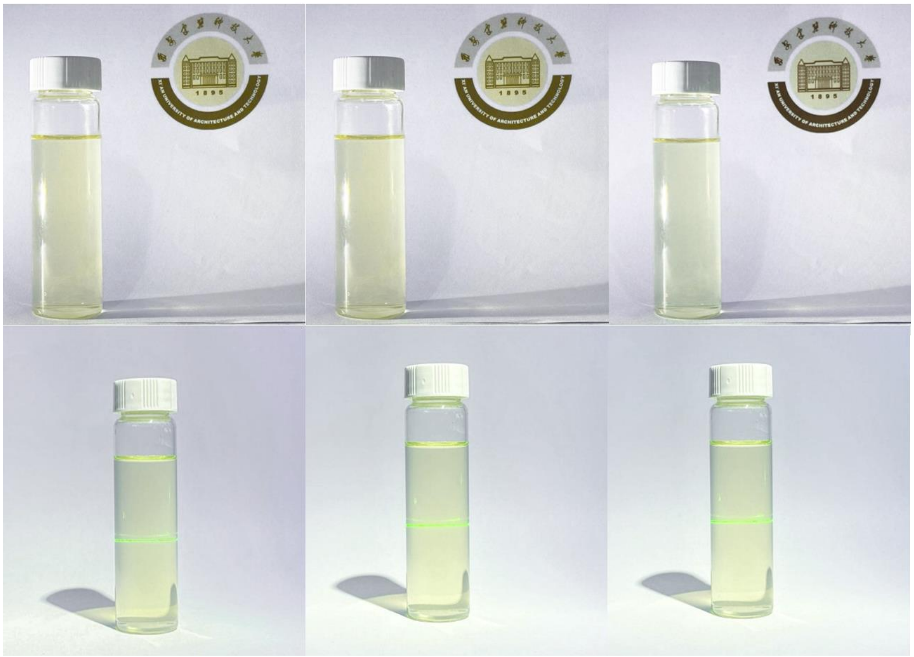

Supplementary Figure 3. The stability of VMT nanosheets dispersion. The VMT nanosheets were well dispersed in water with no significant settling and exhibited a typical Tyndall effect, over a period of 30 days.

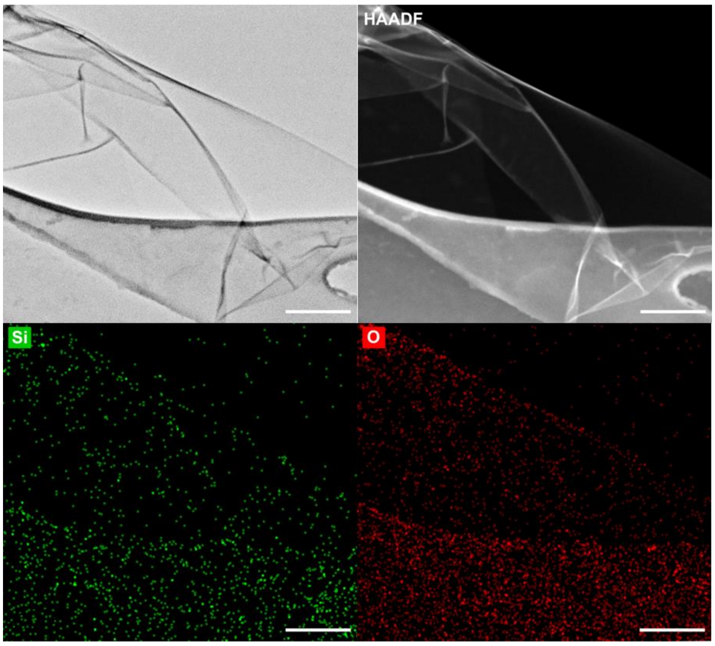

Supplementary Figure 4. TEM images of VMT nanosheet and EDS mappings of oxygen and silicon on the VMT nanosheet. Scale bar, 500 nm.

a b

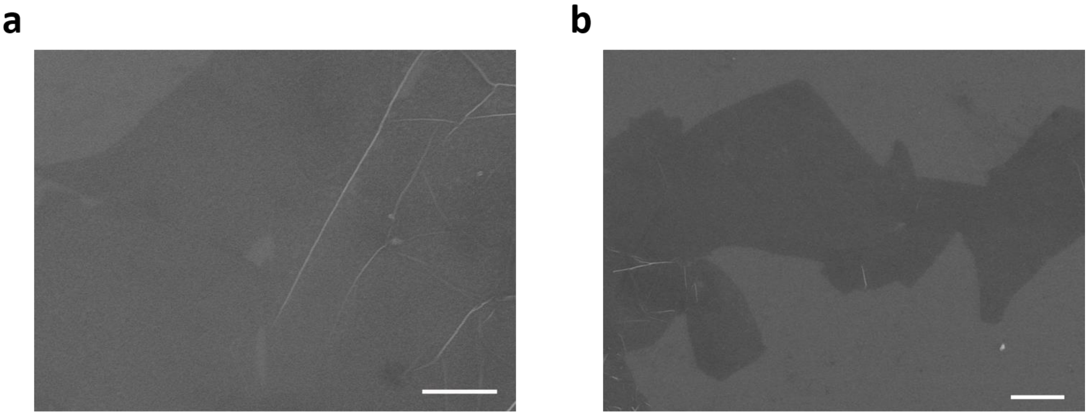

Supplementary Figure 5. SEM image of the VMT nanosheets with different magnifications. Scale bar, 1 μm. **b**, Scale bar, 2 μm.

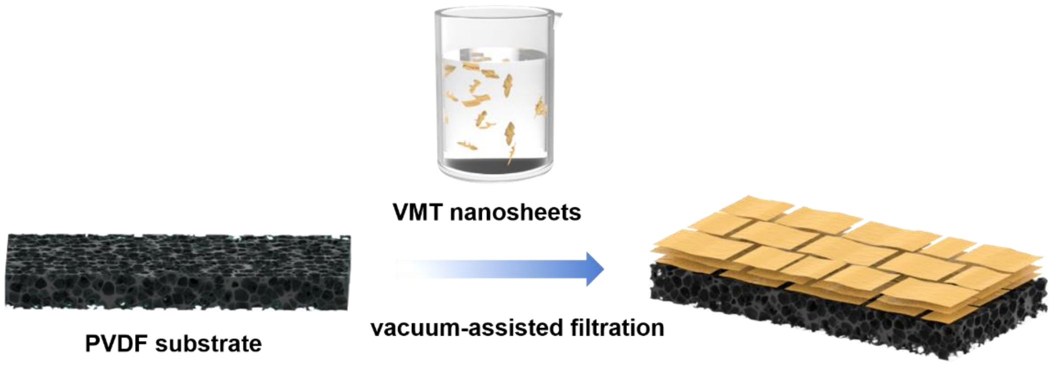

Supplementary Figure 6. Preparation of VMT-based nanochannel membranes. The VMT nanochannel membranes were prepared by parallelly stacking the VMT nanosheets on polyvinylidene fluoride (PVDF) porous substrate using a vacuum-assisted filtration method.

a b

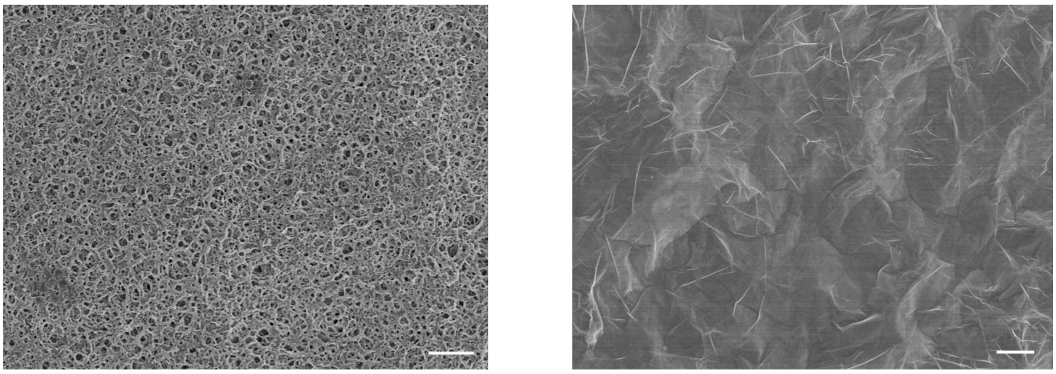

Supplementary Figure 7. Surface SEM images of the membranes. a, Surface SEM image of the PVDF porous substrate. Scale bar, 10 μm. **b**, Top-view SEM image after VMT nanosheets deposition. Scale bar, 2 μm.

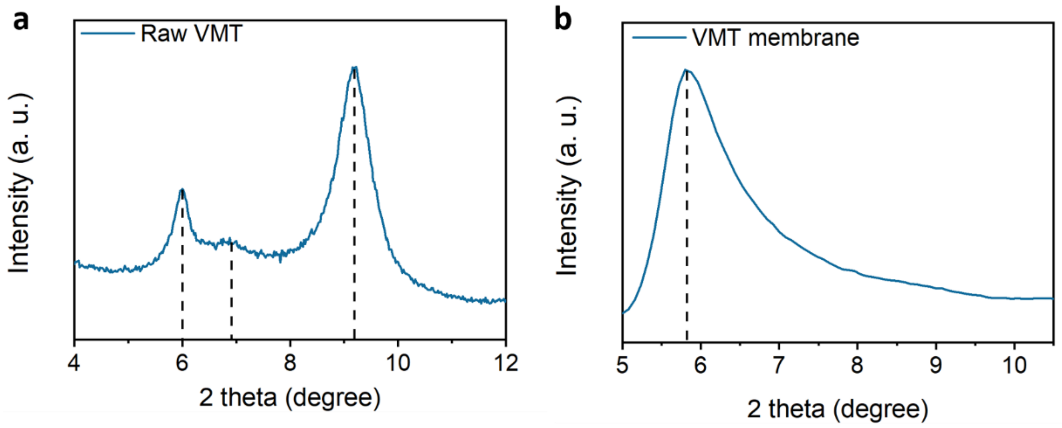

Supplementary Figure 8. XRD images of the VMT. a, XRD pattern of the raw VMT particle. **b**, XRD pattern of the VMT membrane. The three major reflections in the low 2θ range revealed the polycrystalline nature of the raw VMT. And the XRD pattern of the VMT membrane showed a single distinct peak at 5.8°, signifying a d-spacing of 1.53 nm.

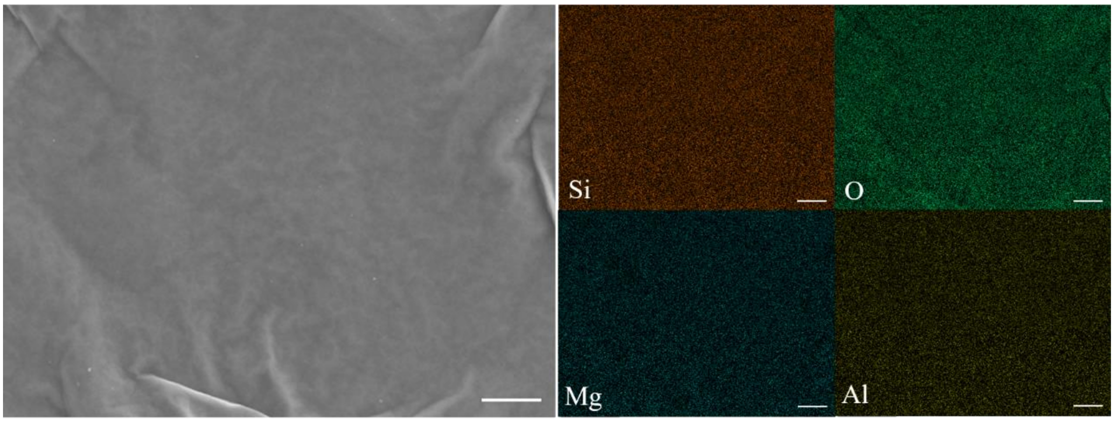

Supplementary Figure 9. Surface SEM mappings of VMT-based nanochannel membranes. Scale bar, 10 μm.

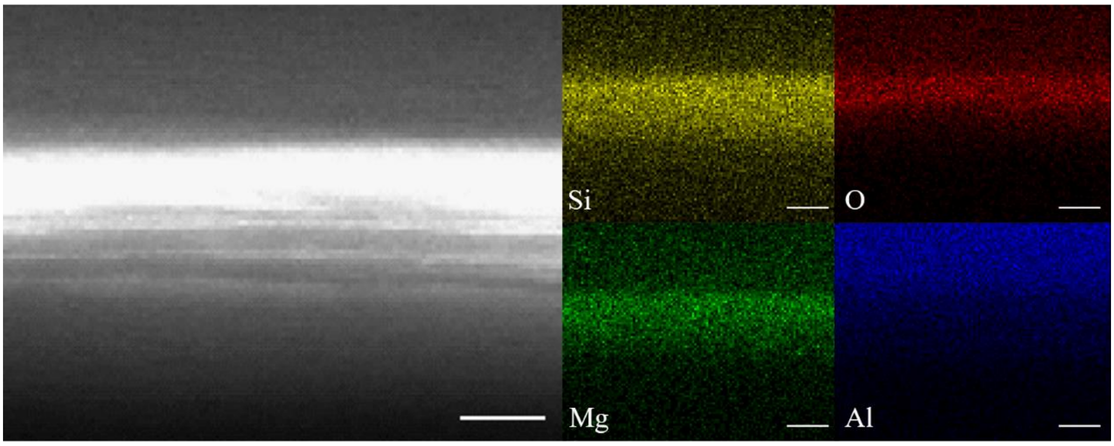

Supplementary Figure 10. Cross-sectional SEM mappings of VMT-based nanochannel membranes. Scale bar, 5 μm.

a b

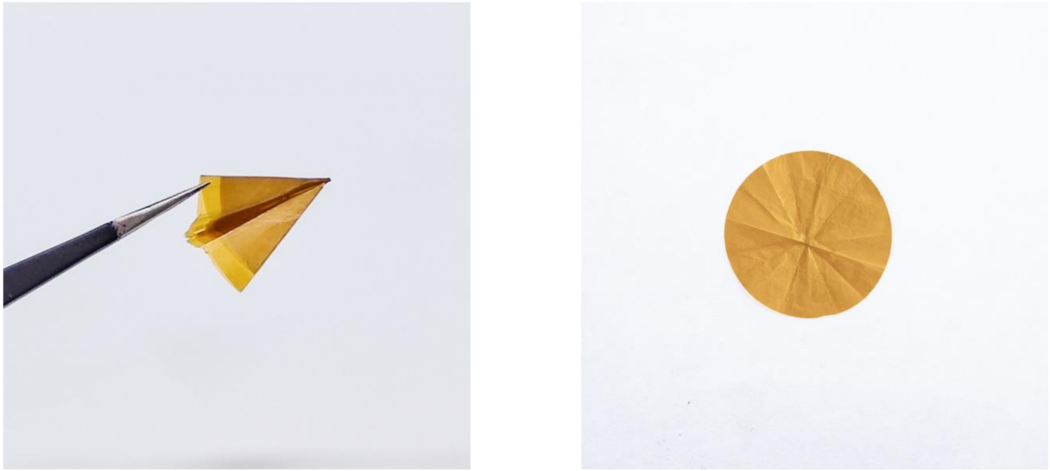

Supplementary Figure 11. Images of the VMT-based nanochannel membrane. a, The VMT-based nanochannel membrane after undergoing multiple folds. **b,** The unfolded VMT-based nanochannel membrane after multiple folding.

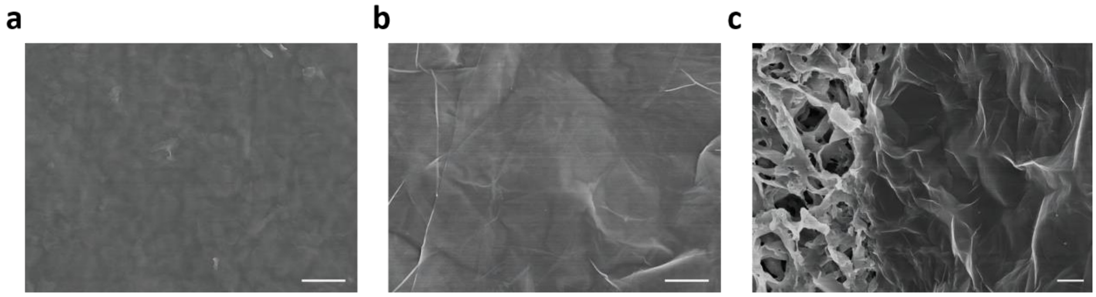

Supplementary Figure 12. Surface morphology SEM images of VMT-based nanochannel membranes. **a**, SEM image of a VMT membrane with the thickness of 2 μm. Scale bar, 1 μm. **b**, SEM image of a VMT membrane with the thickness of 50 nm. Scale bar, 1 μm. **c,** SEM image of a VMT membrane on a PVDF porous substrate. Scale bar, 1 μm. Even though the nanowrinkles increased as the thickness decreasing, the structure of the VMT membrane remained intact and no holes or other defects were visible.

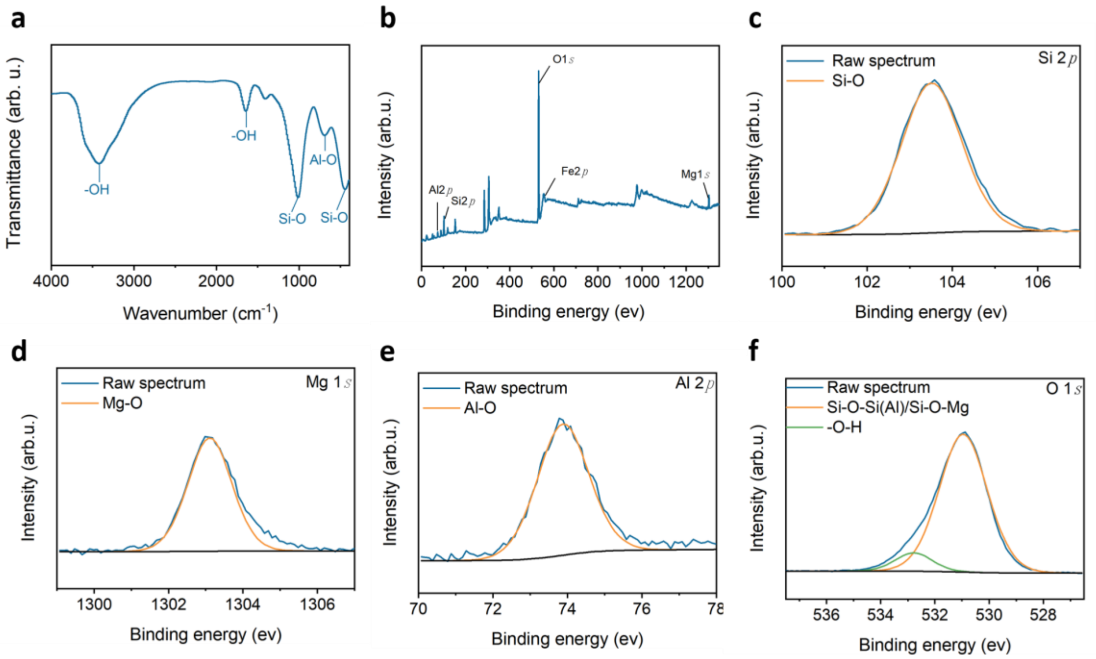

Supplementary Figure 13. FTIR and XPS spectra of the VMT-based nanochannel membrane. a, The peaks observed at 454 and 1000 cm^{-1} could be attributed to the asymmetric stretching vibrations of Si−O. The presence of the absorbance peak at approximately 688 cm^{-1} was assigned to M-O-Si (M = Fe, Al, Mg) plane deformation vibrations, and the broad absorption peaks of near 1640 cm^{-1} and 3400 cm^{-1} were ascribed to –OH vibrations. **b**, XPS spectrum of the VMT membranes. That indicated the presence of Mg, O, Al, Si, which are the main constitutive elements of VMT. **c**, High resolution XPS spectra of Si 2p region. The characteristic peak with bonding energy at 103.5 eV corresponded to Si-O bonds. **d**, High resolution XPS spectra of Mg 1s region. The fitted peak located at 1303 eV binding energy indicated Mg-O bonds. **e**, High resolution XPS spectra of Al 2p region. The presence of Al-O bonds could be inferred from the characteristic peak at 74 eV binding energy. **f**, High resolution XPS spectra of O 1s region. The O 1s peaks at 532.8 eV and 530.8 eV represented the Si-O bonds and the surface hydroxyl groups, respectively.

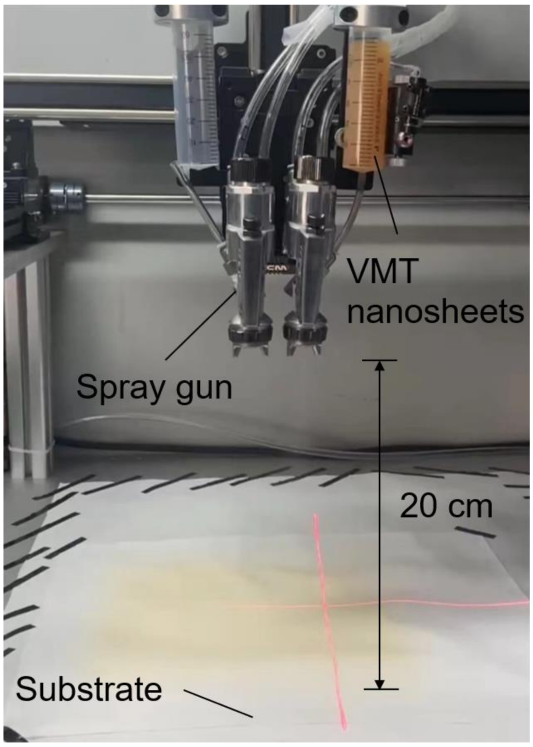

Supplementary Figure 14. Schematic of home-made spray coating device.

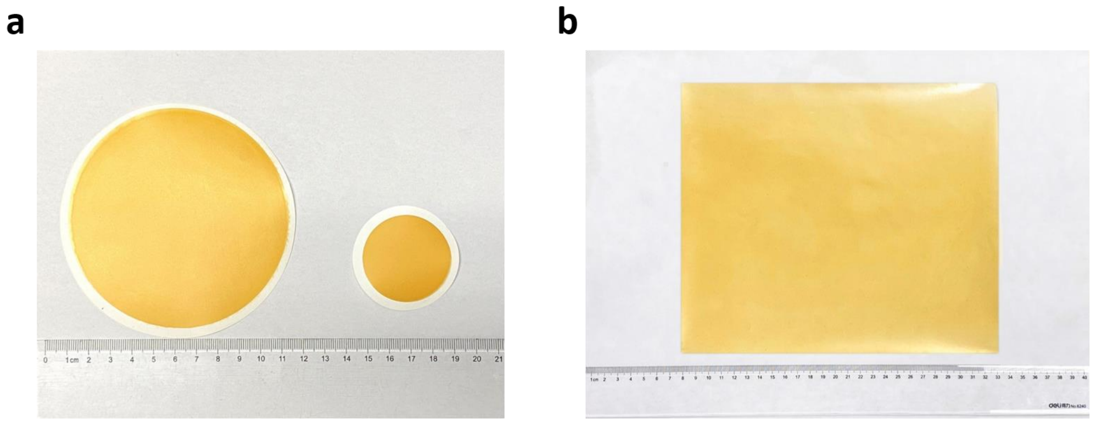

Supplementary Figure 15. Optical images of the VMT-based nanochannel membrane. a, Expansion of membrane area from 12.56 cm^2 to 78.5 cm^2 by vacuum-filtration method. **b,** The membrane covering an area of 300 cm^2 fabricated by spray coating method.

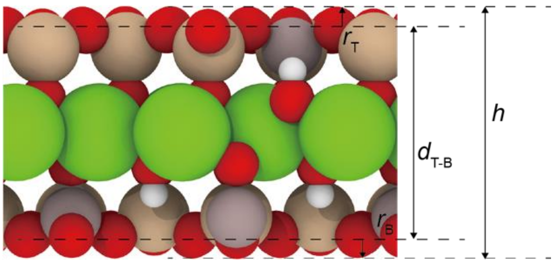

Supplementary Figure 16. The definition of geometric thickness of a monolayer VMT nanosheet.

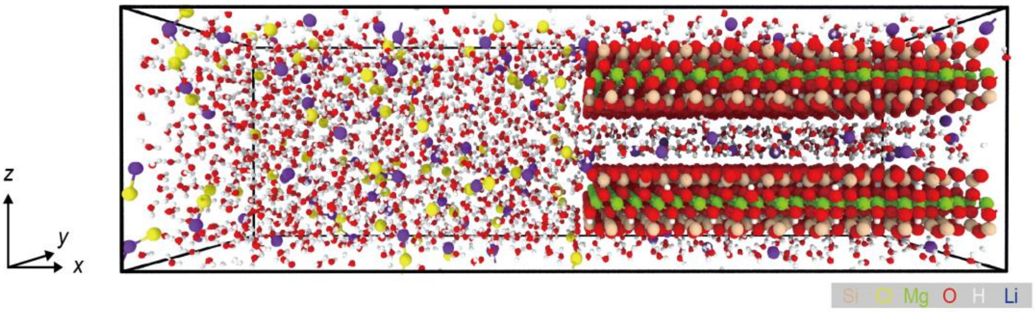

Supplementary Figure 17. The classical MD simulation model.

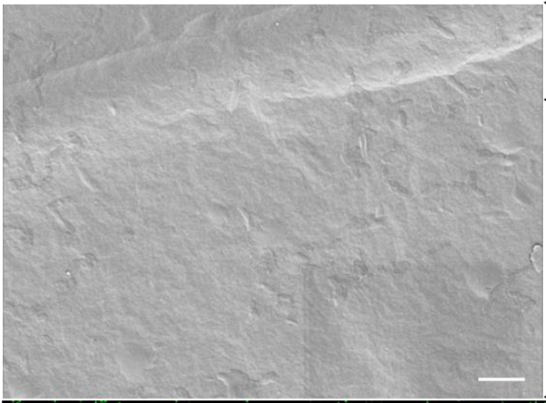

Elements	Atomic%	Weight%
Cl	1.2	1
K	98.8	99
Total	100	100.00

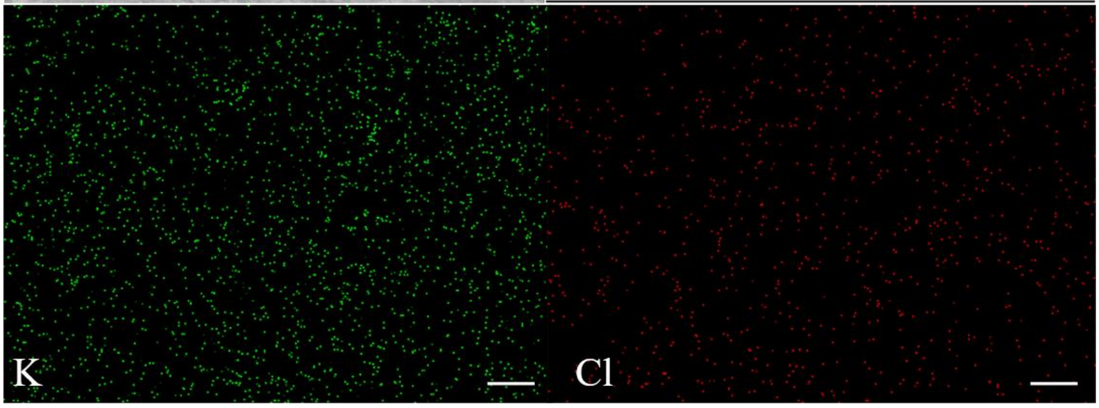

Supplementary Figure 18. EDS mappings of potassium and chlorine on VMT-based nanochannel membrane after immersing in 1 M KCl solution for 10 h. The negatively charged VMT surface showed preferential selectivity for K^+ due to strong electrostatic effect. Scale bar, 5 μm.

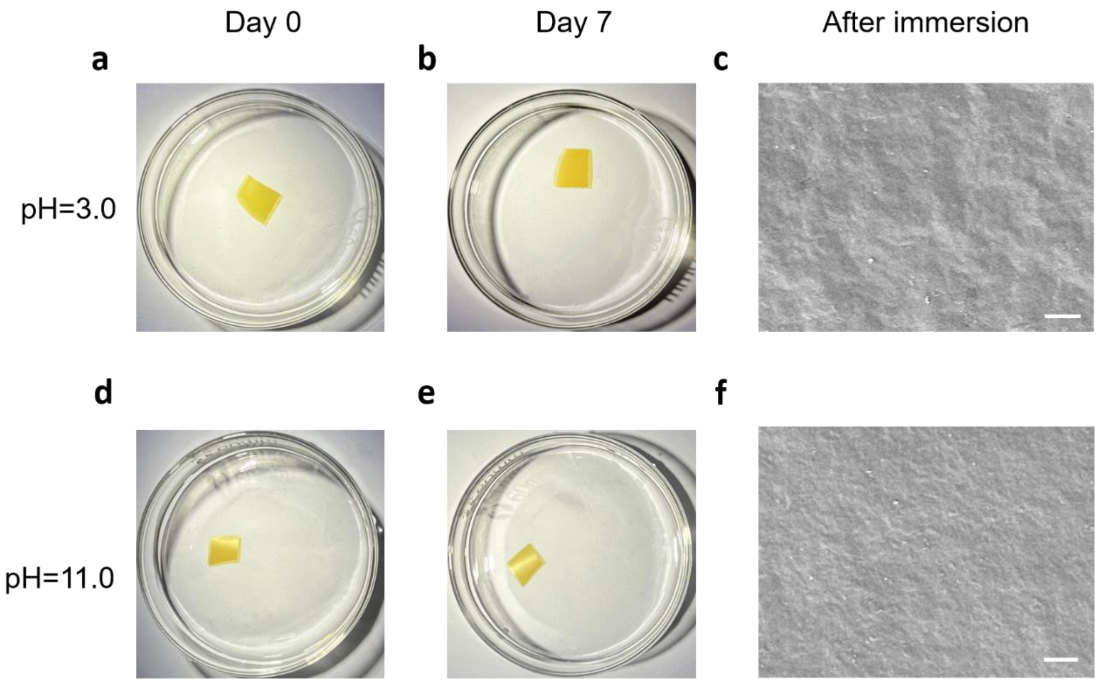

Supplementary Figure 19. The stability of the VMT-based nanochannel membranes in water. a and b, Before and after immersion in water at pH=3.0 for 7 days. **c**, Surface morphology SEM image of VMT membrane after immersion. **d and e,** Before and after immersion in water at pH=11.0 for 7 days. **f**, Surface morphology SEM image of VMT membrane after immersion. Both of them remained structurally intact, with no significant changes in color or surface morphology. Scale bar, 2 μm.

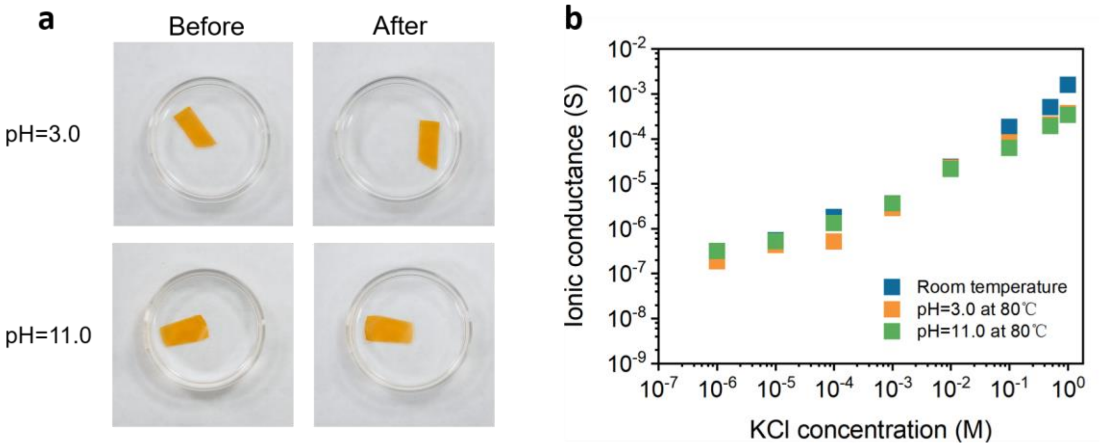

Supplementary Figure 20. The Chemical resistance of the VMT-based nanochannel membranes in water at 80℃. a, Surface morphology of the VMT membrane before and after immersion in water at pH=3.0 and pH=11 for 6 h at 80℃, respectively. **b**, Conductivity of VMT membrane as a function of salt concentration after immersion in water at pH=3.0 and pH=11 for 6 h at 80℃, respectively.

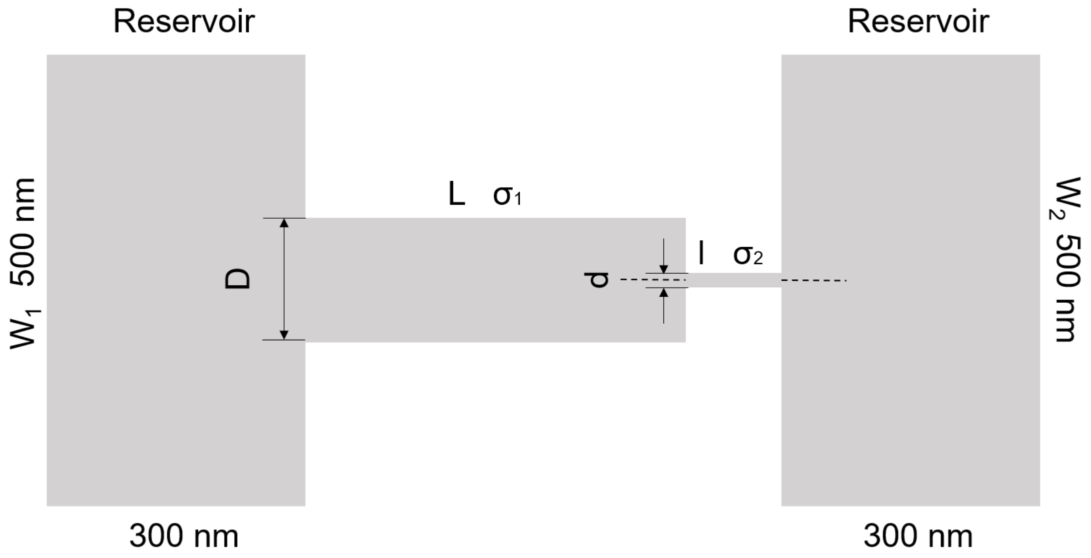

Supplementary Figure 21. Numerical simulation model based on PNP theory.

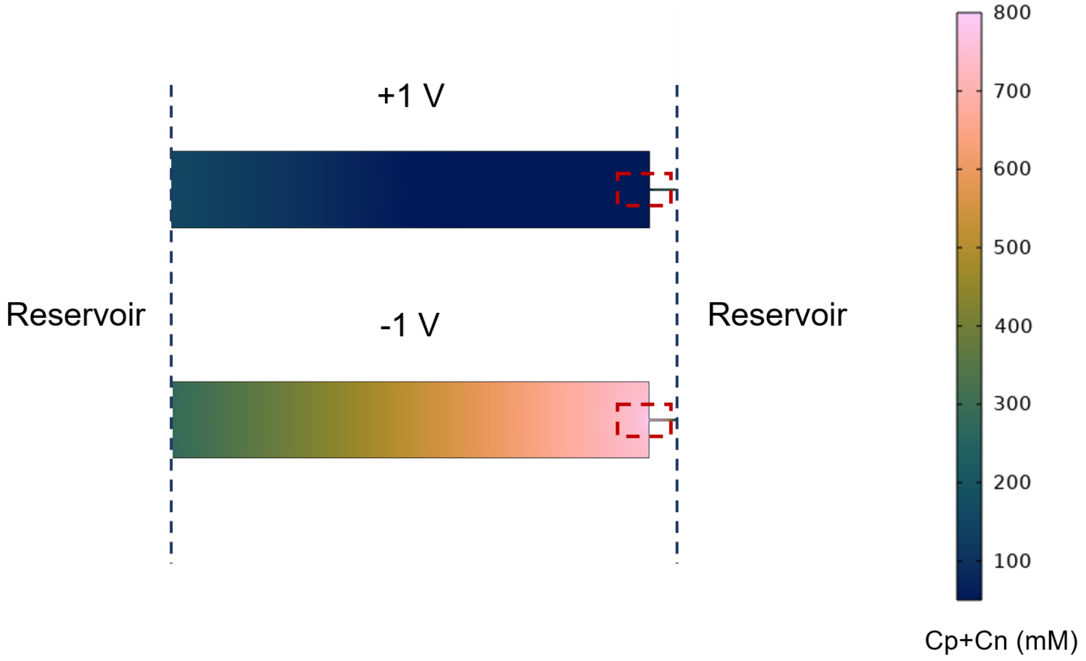

Supplementary Figure 22. Theoretical calculation of ionic concentration distribution in nanochannel at -1 V and +1 V. The results marked in the wireframe section were consistent with that in the main text.

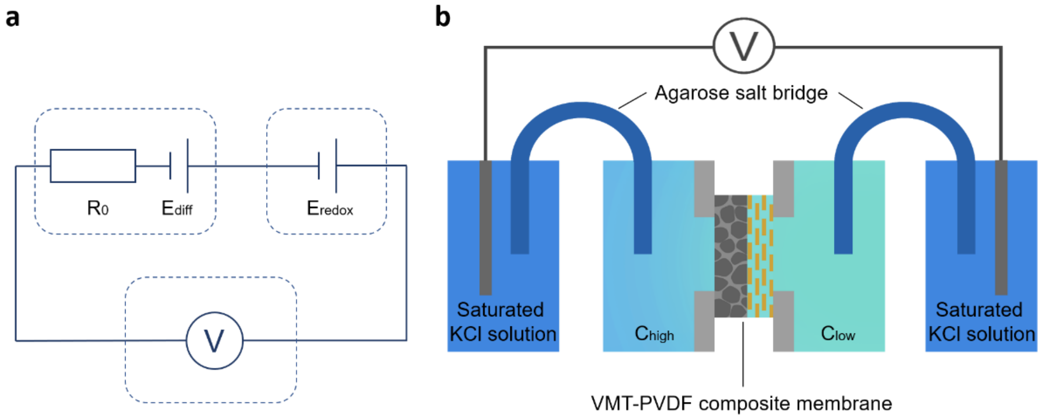

Supplementary Figure 23. Scheme of experimental setups. a, The equivalent circuit diagram of the power generation. **b**, Electrochemical cell for measuring the diffusion potential (E_{diff}) and short-circuit current (I_{SC}) under different gradient. The open-circuit potential (V_{OC}) was comprised of two components, namely, the E_{diff} derived from the membrane and the redox potential (E_{redox}) generated by the unequal potential drop of the electrode-solution interface at different electrolyte concentrations. A pair of agarose salt bridges were used to eliminate the E_{redox} to obtain the actual E_{diff} and I_{diff}.

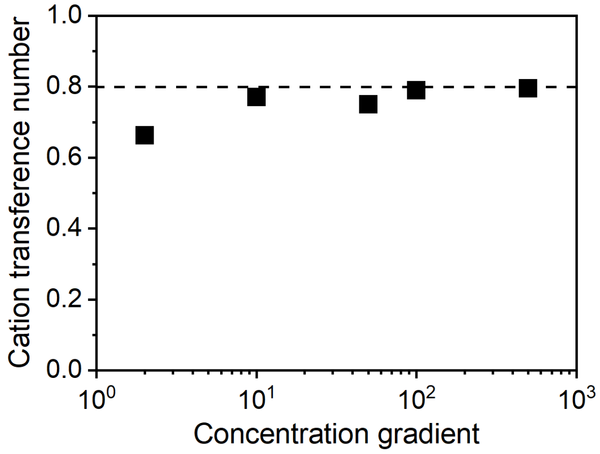

Supplementary Figure 24. Cation transference number (t_+) of the VMT-based nanochannel membrane versus concentration gradient. The calculated t_+ remained steady at 0.8 even when the concentration of the electrolyte solution on the high side reached 1 M.

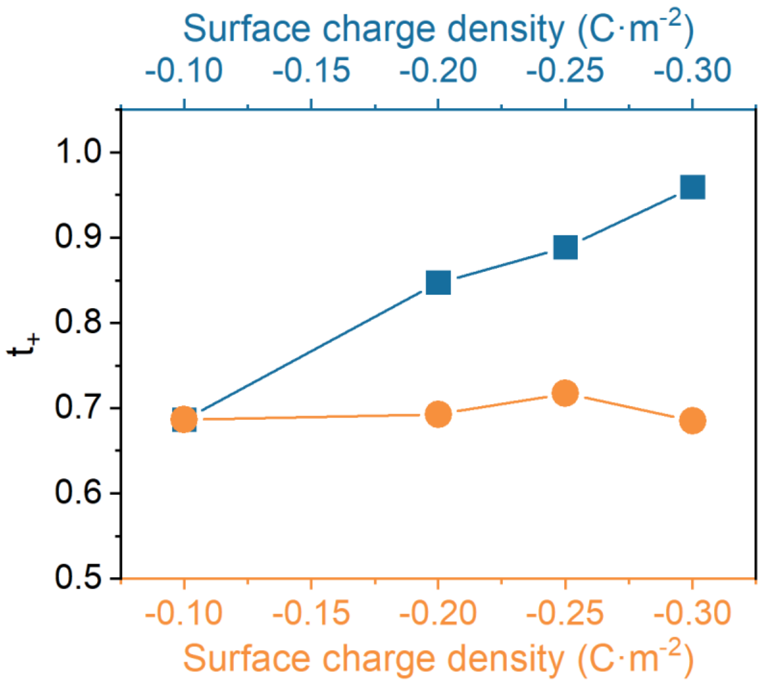

Supplementary Figure 25. The influence of the surface charge density on t_+. When the charge density of the PVDF component was held constant, and the charge density of the VMT layer varied (blue line), it was observed that an increase in the surface charge density of the VMT section resulted in an apparent increase in the t_+. On the contrary, when the charge density of the VMT section was fixed, while changing the charge density of PVDF part (orange line), t_+ was kept at a lower value of about 0.7.

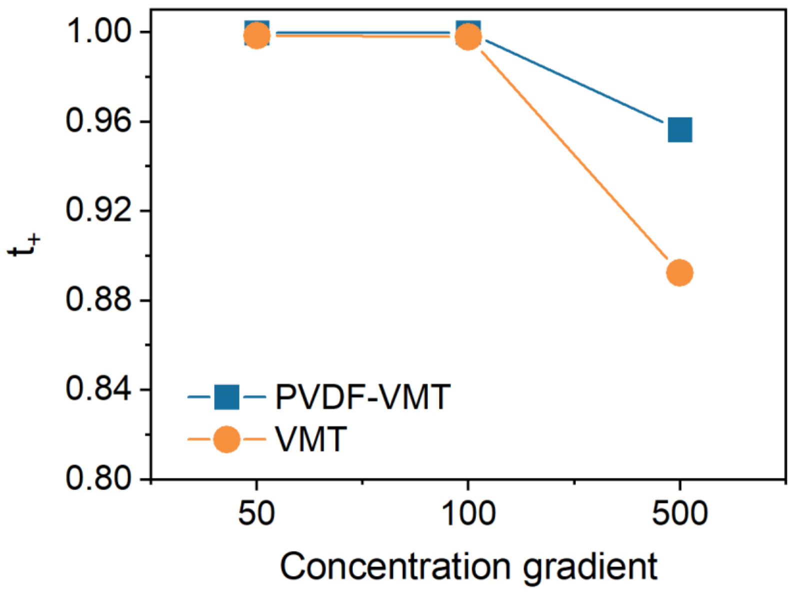

Supplementary Figure 26. The t_+ at different salinity gradients under the two models. The incorporation of the PVDF component in the composite system showed a positive impact on improving the total ion selectivity, and the composite system demonstrated t_+ values exceeding 0.9 at 500-fold salinity gradient compared to a single VMT channel.

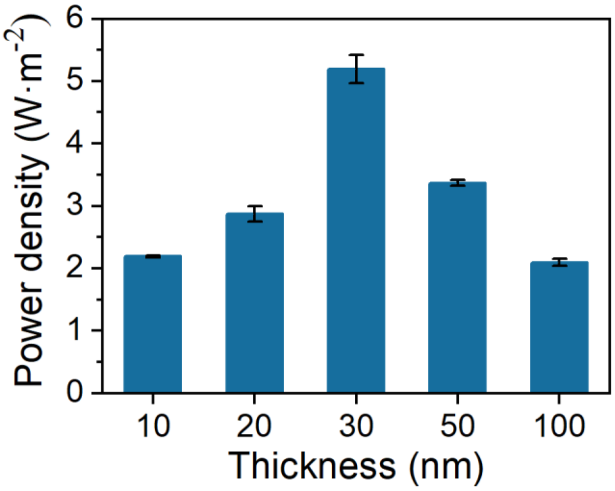

Supplementary Figure 27. Schematically illustrating the impact of VMT layer thickness on osmotic energy generation. The maximum output power density (P_{max}) of 5.45 W m^{-2} was achieved when the ion flux and selectivity effect were balanced at a VMT membrane thickness of 30 nm. The error bars represent the standard deviations of three parallel tests.

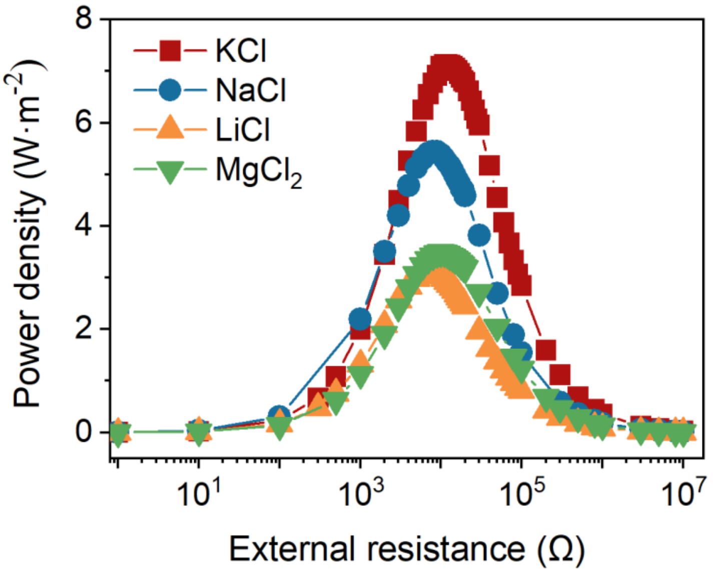

Supplementary Figure 28. The output power density of VMT-based nanochannel membrane as functions of load resistance in different electrolyte solutions. The salt solutions of KCl, NaCl, LiCl, and $MgCl_2$ with the concentration of 0.5 M were used in PVDF side, and 0.01M in VMT side, respectively.

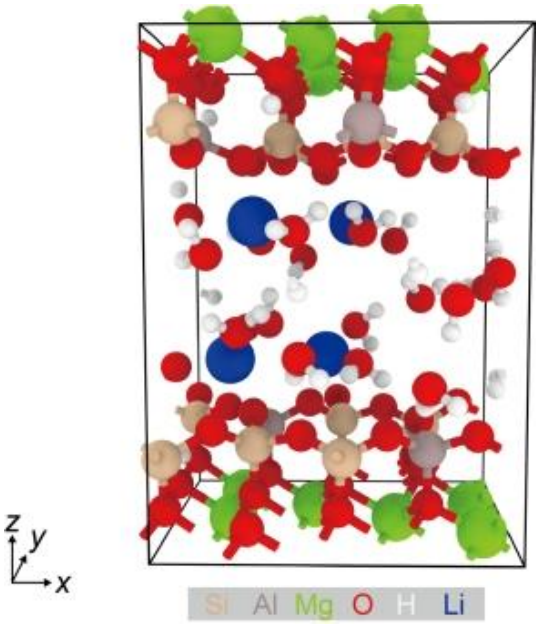

Supplementary Figure 29. The AIMD simulation model that 1/4 of the Si atoms in the basal plane of the VMT structure were substituted with Al atoms, which was the typical structure found in natural VMT. We used an orthorhombic supercell with the size of 10.79×9.35×15.3 Å that contains one-layer VMT and confined water solution.

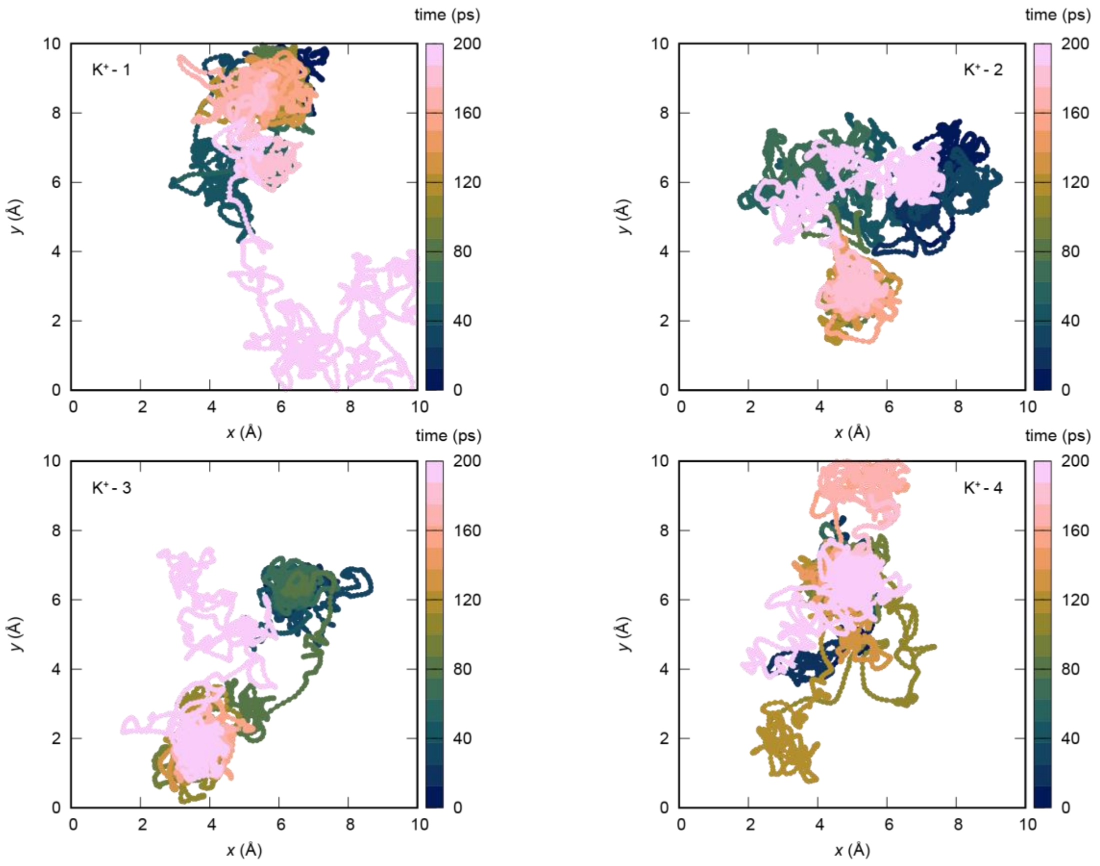

Supplementary Figure 30. The trajectories of 4 K^+ ions in x-y plane. The color of lines meant the diffusion time. The videos of motion of ions were shown in supplementary videos. The results showed K^+ migrated among the center of siloxane rings mainly.

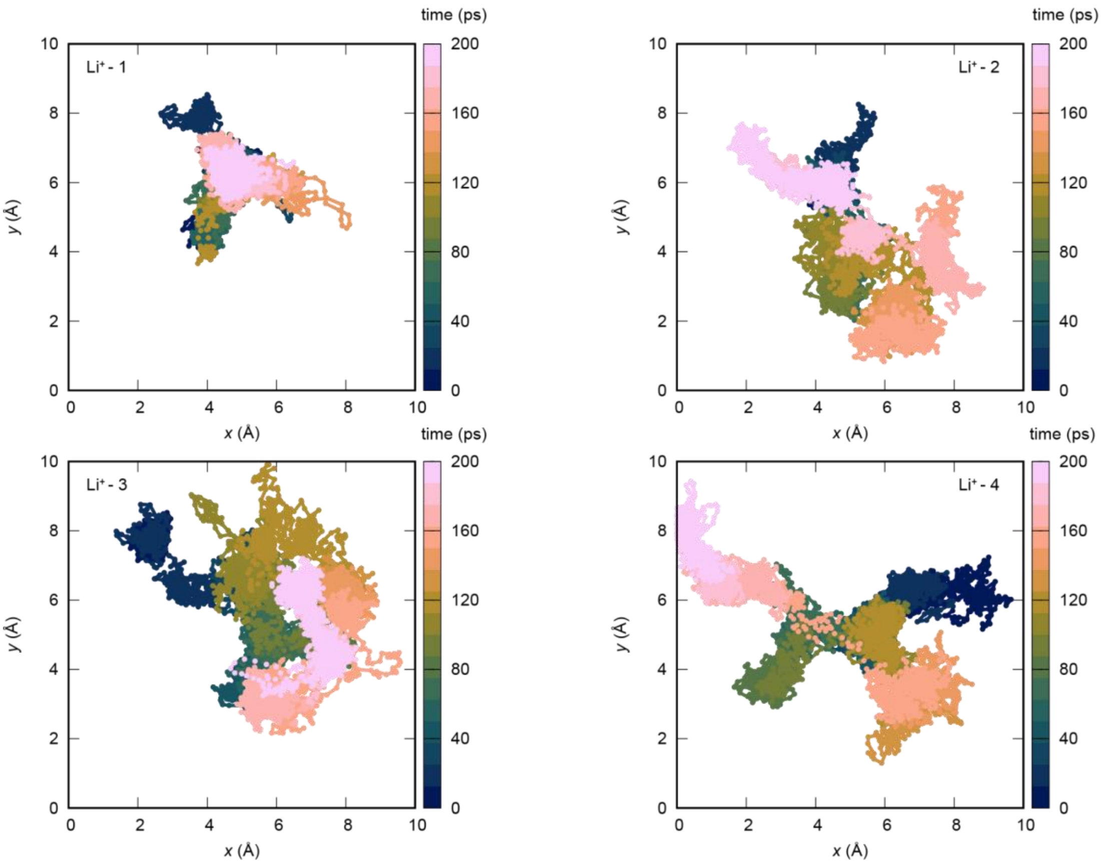

Supplementary Figure 31. The trajectories of 4 Li^+ ions in x-y plane. The color of lines meant the diffusion time. The videos of motion of ions were shown in supplementary videos. The results showed Li^+ migrated near the oxygen groups of siloxane rings.

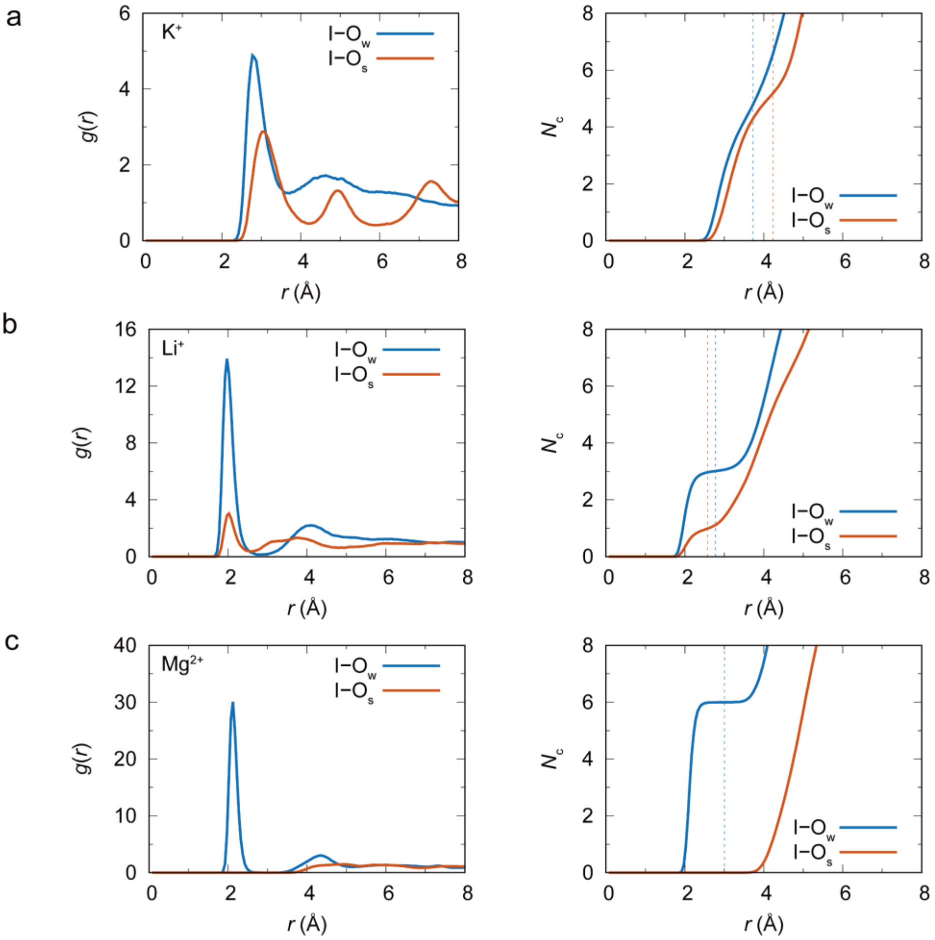

Supplementary Figure 32. The radial distribution function (g(r), RDF, left) between ions and water (O atoms, I-Ow) and oxygen atoms of siloxane rings in VMT nanochannel (I-Os). The coordination number (Nc) curves that mean the number of O atoms around the ions at certain distance were also shown (right), which was equal to the integral of g(r). The dash lines on right figures indicated the boundary of first coordination shell, which was the first minimal on the g(r). **a**, The results for K^+, **b**, Li^+ and **c**, Mg^{2+}. Combining with the results of trajectories (Supplementary Figs. 26 and 27 and supplementary videos), we could get the conclusions as follow. The results showed K^+ and Li^+ would be absorbed near the surface while not for Mg^{2+}. The K^+ migrated among the center of siloxane rings that coordinated with about five O atoms while Li^+ migrated near the oxygen groups of siloxane rings that coordinated with about one O atoms. As for Mg^{2+}, it migrated in the middle of the channel.

Table 1. List of parameters for numerical simulation model.

Parameter	Description	Value	Parameters involved in the manuscript
D	Macropore diameter	80 nm	
L	Macropore length	500 nm	
d	Nanopore diameter	2 nm	
l	Nanopore length	30 nm	
σ_1	Macropore surface charge density	-0.015 C m^{-2}	Fig. 2i, Supplementary Fig. 18
		-0.1 C m^{-2}	Fig. 3c
		-0.25 C m^{-2}	Supplementary Fig. 22
		-0.1 C m^{-2}-0.3 C m^{-2}	Supplementary Fig. 21
σ_2	Nanopore surface charge density	-0.015 C m^{-2}	Fig. 2i, Supplementary Fig. 18
		-0.1 C m^{-2}	Fig. 3c
		-0.3 C m^{-2}	Supplementary Fig. 22
		-0.1 C m^{-2}-0.3 C m^{-2}	Supplementary Fig. 21

Table 2. Comparison with reported osmotic energy conversion performance of nanofluidic membranes.

Materials	P_{max} (W m^{-2}) 0.5M:0.1M NaCl	P_{max} (W m^{-2}) 5M:0.1M NaCl	$P_{max-500}/P_{max-50}$	Refs.
BHMXM	8.6	17.8	2.07	1
SPX membrane	0.62	0.88	1.42	2
TOBC/GO	0.53	0.87	1.64	3
GO/CNFs	4.19	13.25	3.16	4
MXene	0.53	1.1	2.08	5
bsGOM	5.5	18.8	3.42	6
SF membrane	4.06	21.66	5.33	7
KANF membrane	4.8	15	3.13	8
PES-Py/ PEAK-HS	2.6	5.1	1.96	9
SPEEK/AAO/PPy	9.65	26.22	2.72	10
SPEEK membrane	5.8	20.2	3.48	11
AAc/m membrane	4.08	11.72	2.87	12
PIL membrane	4.33	15.46	3.57	13
CPAM	4.8	12.5	2.60	14
CNF/GON	5.26	5.69	1.08	15
HEMAP hydrogel membrane	5.38	20.54	3.82	16
TFP-TPA COF@ANM	5.41	27.8	5.14	17
VMT-based nanochannel	5.45	33.76	6.19	This work

Table 3. The hydration energy (ΔH) of ions in bulk water at ambient conditions[18].

Ions	Hydrated ion diameter (Å)	ΔH (eV)
Li^+	7.64	7.12
Na^+	7.16	6.32
K^+	6.62	5.33
Mg^{2+}	8.56	24.34

Table 4. Composition and concentrations of practical saline brines.

	The composition and concentration of ions in practical saline brines (M)						
	Li^+	Na^+	K^+	Mg^{2+}	Ca^{2+}	Fe^{2+}	Total
Da Chaidam Salt Lake	0.001	3.926	0.106	0.583	0.006	0	4.623
Chaerhan Salt Lake	0.027	3.826	0.203	1.012	0.009	0.001	5.078
Da Yantan Salt Lake	0.017	0.956	0.546	3.045	0.001	0.018	4.583

Supplementary references

1. Ding, L. et al. Bioinspired Ti_3C_2Tx MXene-Based Ionic Diode Membrane for High-Efficient Osmotic Energy Conversion. *Angew. Chem. Int. Ed.* **61**, 41 (2022).
2. Zhu, Q. et al. A sulfonated ultramicroporous membrane with selective ion transport enables osmotic energy extraction from multiform salt solutions with exceptional efficiency. *Energy Environ. Sci.* **15**, 4148-4156 (2022).
3. Sheng, N. et al. TEMPO-oxidized bacterial cellulose nanofibers/graphene oxide fibers for osmotic energy conversion. *ACS Appl. Mater. Interfaces* **13**, 22416-22425 (2021).
4. Wu, Y. et al. Enhanced ion transport by graphene oxide/cellulose nanofibers assembled membranes for high-performance osmotic energy harvesting. *Mater. Horizons* **7**, 2702-2709 (2020).
5. Liu, P. et al. Neutralization reaction assisted chemical-potential-driven ion transport through layered titanium carbides membrane for energy harvesting. *Nano Lett.* **20**, 3593-3601 (2020).
6. Qian, Y. et al. Boosting osmotic energy conversion of graphene oxide membranes via self-exfoliation behavior in nano-confinement spaces. *J. Am. Chem. Soc.* **144**, 13764-13772 (2022).
7. Chen, J. et al. Ultrathin and robust silk fibroin membrane for high-performance osmotic energy conversion. *ACS Energy Lett.* **5**, 742-748 (2019).
8. Ding, L. et al. Ultrathin and ultrastrong kevlar aramid nanofiber membranes for highly stable osmotic energy conversion. *Adv. Sci.* **9**, 25 (2022).
9. Zhu, X. et al. Unique ion rectification in hypersaline environment: A high-performance and sustainable power generator system. *Sci. Adv.* **4**, 10 (2018).
10. Hao, J. et al. A euryhaline-fish-inspired salinity self-adaptive nanofluidic diode leads to high-performance blue energy harvesters. *Adv. Mater.* **34**, 31 (2022).
11. Zhao, Y. et al. Robust sulfonated poly (ether ether ketone) nanochannels for high-performance osmotic energy conversion. *Natl. Sci. Rev.* **7**, 1349-1359 (2020).
12. Chen, W. et al. Improved ion transport in hydrogel-based nanofluidics for osmotic energy conversion. *ACS Cent. Sci.* **6**, 2097-2104 (2020).
13. Hu, Y. et al. Bioinspired poly (ionic liquid) membrane for efficient salinity gradient energy harvesting: Electrostatic crosslinking induced hierarchical nanoporous network. *Nano Energy* **97**, 107170 (2022).
14. Hou, S. et al. Charged porous asymmetric membrane for enhancing salinity gradient energy conversion. *Nano Energy* **79**, 105509 (2021).
15. Zhao, Y. et al. Cement-and-pebble nanofluidic membranes with stable acid resistance as osmotic energy generators. *Sci. China Mater.* **65**, 2729-2736 (2022).
16. Chen, W. et al. Improved ion transport and high energy conversion through hydrogel membrane with 3D interconnected nanopores. *Nano Lett.* **20**, 5705-5713 (2020).
17. Gao, M. et al. A bioinspired ionic diode membrane based on sub-2 nm covalent organic framework channels for ultrahigh osmotic energy generation. *Nano Energy* **105**, 108007 (2023).
18. Marcus, Y. *Ions in Solution and their Solvation*. (John Wiley & Sons. Press, 2015).
