# Supplementary material for: Unlocking osmotic energy harvesting potential in challenging real-world hypersaline environments through vermiculite-based hetero-nanochannels
Source: Nat Commun. 2024 Jan 19;15:608. doi: 10.1038/s41467-023-44434-1 (PMC10799064; doi:10.1038/s41467-023-44434-1)
Supplement: Supplementary file 3 — Description of Additional Supplementary Files [file 41467_2023_44434_MOESM3_ESM.pdf]

Description of Additional Supplementary Files

File Name: Supplementary Data 1
Description: Computational model for AIMD simulation.

File Name: Supplementary Data 2
Description: Initial and final configurations for classical MD simulation.

File Name: Supplementary Movie 1
Description: The diffusion path of K+ migrating among the centre of siloxane rings.

File Name: Supplementary Movie 2
Description: The diffusion path of Li+ migrating near the oxygen groups of siloxane rings.
